# Antiobesity Effect of Caraway Extract on Overweight and Obese Women: A Randomized, Triple-Blind, Placebo-Controlled Clinical Trial

**DOI:** 10.1155/2013/928582

**Published:** 2013-11-10

**Authors:** Mahnaz Kazemipoor, Che Wan Jasimah Bt wan Mohamed Radzi, Majid Hajifaraji, Batoul Sadat Haerian, Mohammad Hossein Mosaddegh, Geoffrey A. Cordell

**Affiliations:** ^1^Department of Science & Technology Studies, Faculty of Science, University of Malaya, 50603 Kuala Lumpur, Malaysia; ^2^National Nutrition & Food Technology Research Institute, Faculty of Nutrition & Food Technology, Shahid Beheshti University of Medical Sciences, Tehran 1981619573, Iran; ^3^Department of Pharmacology, Faculty of Medicine, University of Malaya, 60302 Kuala Lumpur, Malaysia; ^4^Department of Pharmacology and Toxicology, Pharmacy School, Yazd Shahid Sadoughi Medical Sciences University, Yazd 8917945556, Iran; ^5^Natural Products Inc., Evanston, IL 60203, USA

## Abstract

Caraway (*Carum carvi* L.), a potent medicinal plant, is traditionally used for treating obesity. This study investigates the weight-lowering effects of caraway extract (CE) on physically active, overweight and obese women through a randomized, triple-blind, placebo-controlled clinical trial. Seventy overweight and obese, healthy, aerobic-trained, adult females were randomly assigned to two groups (*n* = 35 per group). Participants received either 30 mL/day of CE or placebo without changing their diet or physical activity. Subjects were examined at baseline and after 90 days for changes in body composition, anthropometric indices, and clinical and paraclinical variables. The treatment group, compared with placebo, showed a significant reduction of weight, body mass index, body fat percentage, and waist-to-hip ratio. No changes were observed in lipid profile, urine-specific gravity, and blood pressure of subjects. The results suggest that a dietary CE with no restriction in food intake, when combined with exercise, is of value in the management of obesity in women wishing to lower their weight, BMI, body fat percentage, and body size, with no clinical side effects. In conclusion, results of this study suggest a possible phytotherapeutic approach for caraway extract in the management of obesity. This trial is registered with NCT01833377.

## 1. Introduction

Proper nutrition is necessary to keep the body healthy and functioning normally. The addition of extra calories in the diet induces fat accumulation, leading to overweight and obesity. According to the World Health Organization (WHO), excess body weight and obesity are recognized as a body mass index (BMI) greater than 25 kg/m^2^. According to this report by WHO, “globesity,” as a foodborne illness, is a rapidly growing global problem, which is maximizing the risk of various health problems, such as type 2 diabetes, cardiovascular diseases (CVD), musculoskeletal disorders, and cancer. Overweight and obesity are associated with high morbidity and mortality, resulting in considerable health care costs and other economic and social impacts on the society. Since 1980, obesity has almost doubled worldwide and is recognised as one of the leading causes of death. In 2008, over 1.4 billion adults, predominantly women, were overweight or obese. Finally, more people die because of being overweight and obese than those who are underweight, and this disease state is the fifth main reason for mortality and the sixth for health problems globally. Management of obesity is therefore a public health necessity [1–5].

Obesity is associated with multiple macro- and microenvironmental factors. It is manageable by several different approaches, including pharmaceutical drugs, traditional medications, and surgery. Of these approaches, the use of medicinal plants is increasingly popular and is preferable to conventional chemotherapeutic methods [6, 7]. Early indigenous people faced with various forms of illness and health problems discovered a wealth of valuable healing agents in their local flora. There are a frequently number of potential advantages linked to the use of medicinal plants, including accessibility, safety, effectiveness, affordability, reliability, and acceptability, typically with minor adverse effects and lower costs [8]. Moreover, medicinal plants are naturally available sources with potentially beneficial biological and pharmacological effects and are easy to consume, whereas there is still doubt about the other more invasive therapeutic modalities for obesity, such as surgery [9]. 

Specific phytochemical constituents present in medicinal plants may assist in regulating weight and body fat through modifying metabolic pathways at the molecular level which are responsible for signalling adipogenesis, lipolysis, and so forth [10]. Hence, they are able to play multiple roles simultaneously in a network pharmacology approach to disease alleviation. 

Caraway (*Carum carvi* L.) is a well-known medicinal plant, which was traditionally recommended by the great ancient scientist of the eleventh century Ibn Sina (also known as Avicenna) for weight loss, and is used widely for culinary purposes in Asia and Europe [11]. This plant is from the Apiaceae (formerly Umbelliferae) family and is used in traditional medicine as a remedy for a range of health problems, especially stomach ache, burping and flatulence, and intestinal spasms [12–17]. Caraway seeds contain multiple phytochemical constituents, including fatty acids, essential oils, and volatile phenolic compounds which are used in industry and medicine [18–22]. These bioactive ingredients present in caraway seeds induce a range of different biological benefits, including antimicrobial, antioxidant, anti-inflammatory, and anticancer activities, which offers promising therapeutic potential to alleviate several human diseases [23–29].

Previous studies have established an association between the moderate consumption of caraway-derived metabolites with a lower incidence of diabetes, dyslipidaemia, hypertension, liver dysfunction, reproductive hormone imbalance, osteoporosis, cancer, gastrointestinal, and inflammatory diseases [13, 30]. In addition, *in vitro* and *in vivo* studies have demonstrated the hepatoprotection and safety of caraway ingredients for use in pharmaceuticals and food products [30–32]. A further study showed a plausible, multitargeted, antiobesity effect of caraway on animals through modifying the gene expressions associated with inflammation and adipogenesis [33]. Accordingly, this activity of caraway was examined in a clinical study of overweight and obese subjects as a dietary intervention, in combination with physical activity, in a homogenous population of physically active, adult women selected to evaluate the reliability of the earlier indications in a formal clinical environment. 

## 2. Methods

### 2.1. Study Design and Study Population

The clinical study reported was a randomized, triple-blind, placebo-controlled, clinical trial, with a duration of three months, and was designed and conducted to evaluate the weight-lowering effect of the caraway seed extract (CE) compared with placebo. Obese and overweight women with a BMI (body mass index) of 25–39.9 kg/m^2^ and ages between 20 and 55 years were eligible for this study. Volunteers were recruited at a fitness centre in Yazd, Iran, and were doing moderate aerobics training for 180 minutes/week, with an estimated energy expenditure of 1000–1200 kcal/week.

Subjects with a history of extreme weight loss through surgery or abnormal diet and the presence of diagnosed, severe health problems including hypertension, CVD, dyslipidaemia, clinical depression, diabetes mellitus, and thyroid diseases, using alcohol, cigarette, or any medication or supplements which might have an effect on metabolism or appetite, having a history of allergy to the medicinal plant extract or placebo products, and also pregnant and lactating women were specifically excluded from the study cohort. This interventional study was registered with the clinicaltrial.gov protocol registration system with the Protocol no. NCT01833377 and was approved by the Medical Ethics Committee of the University of Malaya Medical Centre (UMMC) on June 20, 2012 (no. 925/15). All of the entered subjects signed an approved, written consent form at the initiation of the study.

Nutritional consultation was provided at baseline entry into the protocol and during the treatment regimen. Participants were encouraged to follow a healthy lifestyle habit and were advised not to make any significant changes to their diet and routine physical activity during the three-month period of the study protocol. 

### 2.2. Randomization and Blindness

The seventy (70) qualified and allotted subjects were randomized into two equal-sized groups of 35-35 subjects through the online randomization program (http://www.randomization.com/). Investigators, subjects, and the data collectors were masked to the treatment regimens. A statistician, who was not directly involved in the establishment of the groupings and the design of the trial, was provided with the codes and the data for analysis (triple-blind). The bottled CE and placebo samples were coded by the coinvestigator who was not involved in the study, and the sample of CE (500 mL) or placebo was provided for the subjects in sealed PET bottles every two weeks. The eligible study subjects were randomly allocated to consume either a 30 mL sample of the active CE or placebo product, once a day, 20 minutes before lunch for 3 months.

### 2.3. Preparation of Herbal Extract and Placebo

The CE samples obtained from the Baharan Company, Yazd, Iran (Industrial Ministry License no. 28/1232 and Health Ministry License no. 35/10500) were extracted from the seeds of caraway through steam distillation. From each 1 kg of caraway seeds, 10 litres of caraway water extract was produced. Consequently, the amount of caraway in terms of w/v was 0.1 (10%). The analysis of the CE sample used in the study is described below. The placebo was prepared by dissolving edible caraway essence (Givaudan Flavours Co., Kemptthal, Switzerland) in drinking water (1% g/L) which was identical with CE in appearance and flavour. Subjects were provided with measured bottles and were asked to dissolve 30 mL of the placebo or CE with 30 mL of water. Subjects were provided with brochures with written instructions. 

### 2.4. Assessments and Study Outcomes

The study visits were conducted two weeks before the beginning of the trial, at the beginning of the trial (week 0), and every week up to the end of the three months of treatment. The screening and data collection were performed by the investigators and a medical physician. During the intervention, all participants were examined and checked weekly to ensure that the study instructions were being followed, and that intake of samples was occurring according to the regimen. The occurrence of any probable side effects was recorded by the volunteers.

Body weight loss was the primary study outcome. The secondary outcomes included changes in body composition (body fat, body water, and body muscle percentages), anthropometric indices (BMI, height, waist circumference, hip circumference, mid-upper arm circumference, and thigh circumference), serum lipid profile, urine-specific gravity, systolic and diastolic blood pressure, and pulse rate. Safety outcomes also included laboratory assessments and vital signs. In addition, the occurrence of adverse events which might be related to the treatment was identified by the investigator and the physician through physical examination. All measurements were assessed early in the morning with an empty stomach and were performed at baseline and at week 12.

Body weight was measured within 0.1 kg intervals. Participants were weighed in light clothing and without shoes using a bioelectrical impedance analysis (BIA) machine with remote control (Beurer digital diagnostic scale, Model BG63, Ulm, Germany). Other body composition parameters including percentages of body fat (%BF), body water (%BW), and body muscle (%BM) were displayed with 0.1% graduation. BMI (kg/m^2^) was calculated based on the folowing formula BMI = weight/height^2^. 

Anthropometric indices including height, waist circumference (WC), and hip circumference were measured to the nearest 0.1 cm, using Seca measuring tape. The waist circumference was measured by placing the measuring tape at the umbilicus point (the site between the lowest rib and the iliac crest); hip circumference (HC) was measured at the maximum circumference over the buttocks (WHO, 2008). Waist-to-hip ratio (WHR) was then calculated by dividing the waist and hip circumferences. 

### 2.5. Clinical Assessments

Vital parameters, including blood pressure and heart rate, were measured by a physician using a calibrated mercury sphygmomanometer, stethoscope, and appropriate cuff sizes on the sitting subject's right arm after a 10 min rest. Systolic, as well as diastolic, blood pressure was defined according to phase I and phase V Korotkoff sounds, respectively. Blood and urine tests were conducted at the reference laboratory of Shahid Sadoughi Hospital in Yazd, Iran in the fasting condition. The biochemical parameters were analysed using the ELITech diagnostics kits (ELITech Group, Puteaux, France).

### 2.6. Sample Size Calculation

The required sample size was calculated using the sample size formula described by Greenberg et al. [34] with 99% level of confidence, 1% precision, and with a power level of 90%. The primary variable was weight, and the sample size was based on a two-tailed *t* test. The standard deviation of weight in the study population was anticipated to be 14 kg, which is similar to the weight measurements obtained from previous studies. According to this formula, a total sample of 60 subjects (30 subjects in each group) was required. To enhance the power for identifying significant differences in weight loss of participants from baseline compared to the control group and assuming dropouts and loss to followup during the three-month study intervention period, 10 extra patients were randomized and included. Hence, a total of seventy (70) overweight and obese women with BMI > 25 were recruited for this study.

### 2.7. Gas Chromatography-Mass Spectrometry (GC-MS) Analysis

The phytochemical constituents present in CE were identified using GC-MS analysis with flame ionization detector (FID) and extracted by HS-SPME with subsequent hexane extraction. The capillary gas chromatographic profiles of the CE constituents were reported as their retention time compared with the MS of standard compounds [35].

### 2.8. Statistical Analysis

Values for each subject were standardized for each dependent variable to remove outliers using Z-scores, and the normal distribution was tested using the Kolmogorov-Smirnov test. Student's *t*-test, with a 99% confidence interval, was applied to identify the significant differences in values between groups, and the paired *t*-test was used to examine mean differences within each group during the three-month treatment period [36]. All statistical analyses were performed using SPSS software version 18.0.0 (SPSS Inc., Chicago, IL, USA), and all data are expressed as mean ± standard deviation (SD); *P* values less than 0.01 were considered to be significant and equal variances were assumed.

## 3. Results

### 3.1. Demographic and Baseline Features of the Subjects

Of the 110 overweight women who originally registered for screening, seventy were deemed eligible to have met the study requirements and constraints. The selected subjects were randomised and assigned equally to the CE and placebo groups. Of the selected overweight women, ten of the subjects—six in the placebo group and four in the CE group-failed to complete the study. At the termination of the study, therefore, sixty of the seventy patients completed the full three months of treatment. The demographic characteristics of the subjects in the study are summarized in Table 1. About 54% of the subjects were overweight and 46% were obese. The mean (SD) age, body weight, and BMI of the participants were 37.11 (8.6) years, 75.43 (11.7) kg, and 29.82 (4.1)  kg/m^2^, respectively. The average physical activity level of the participants was 44 (2.6) (kcal/kg/day) with 7.86 (1.4) hours of sleep. No significant differences of all of these features were observed between the two groups. At baseline, there were no significant differences in the body composition, anthropometric indices, clinical and para-clinical assessments of both groups, except for the waist circumference which was at the borderline. All of the participants had abdominal obesity (waist circumference > 88 cm) (Table 2). 

### 3.2. Comparison within and between Groups during the Three-Month Trial

The changes in variables over the three-month intervention period for the CE and the placebo groups are also shown in Table 2. Significant mean weight loss was observed within the CE group after three months of treatment, whereas the average weight in the placebo group was increased. The mean weight loss between the CE group and placebo group was significant. The mean weight in the CE group dropped remarkably as compared with the placebo group. Therefore, this traditional medicine extract probably has a positive effect on lowering body weight. Similarly, the average BMI and %BF in the CE group were significantly decreased. In contrast, in the placebo group, these values increased slightly. Nevertheless, the mean reduction in the BMI and %BF was significant between the CE group and the placebo group.

The percentage of body muscle in the CE group showed a significant increase after intervention. Body water percentage decreased in both groups and was not significant either within or between groups. On the other hand, the WC and WHR were reduced significantly only in the CE group. However, the level of reduction (cm) in all of the anthropometric indices was remarkable between groups. There were no noticeable changes in para-clinical and clinical assessments with either treatment. According to the outcomes of the study, CE showed greater efficacy than did placebo for each primary outcome measure.

### 3.3. Safety Issues and Adverse Events

No significant changes were observed for heart rate, systolic and diastolic blood pressure, lipid profile, and urine-specific gravity between and within the two groups during the three-month study period. Of the sixty subjects who completed the study, only the placebo participants experienced skin allergy to the placebo product, and no important adverse events were reported during the physical examinations. 

### 3.4. Detection of Phytochemicals Using GC-MS

**Figure 1:**
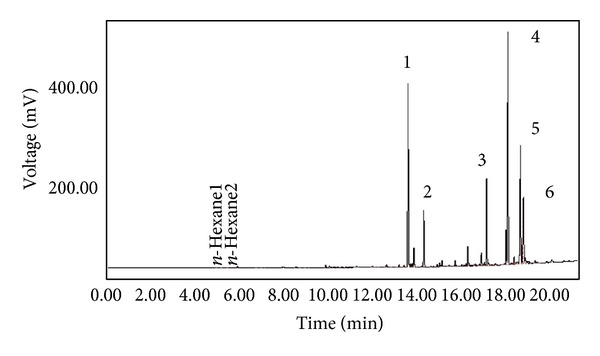
Chromatogram of CE infusion extracted by HS-SPME. Identification of CE volatiles obtained by steam distillation with subsequent hexane extraction, including (1) limonene, (2) *γ*-terpinene, (3) *trans*-carveol, (4) carvone, (5) thymol, and (6) carvacrol.

The principal volatile compounds analyzed by GC-MS following extraction were mostly monoterpenoids, as shown in the chromatogram in Figure 1.

## 4. Discussion

The weight-lowering property of caraway as a known medicinal plant in Iran was examined in a triple-blind, placebo-controlled, clinical trial in Iranian overweight and obese women. Since diet and physical activity are the two lifestyle principles which induce normal weight, subjects were selected who were regularly performing aerobic exercises during the entire period of study without modifying their diet and lifestyle habits. The results indicated a moderate effect of CE on losing weight, without any severe adverse effects. This finding is consistent with a recent study which reported data of antiobesity effect of this plant in an animal model [33]. Additionally, numerous studies have reported the therapeutic effects of caraway on different diseases such as diabetes mellitus, cardiovascular disease (CVD), and hypertension, which are known as common complications of obesity [37–39]. Altogether, the results of this study suggest a plausible phytotherapeutic approach for the use of caraway seed extract in the management of obesity.

**Figure 2:**
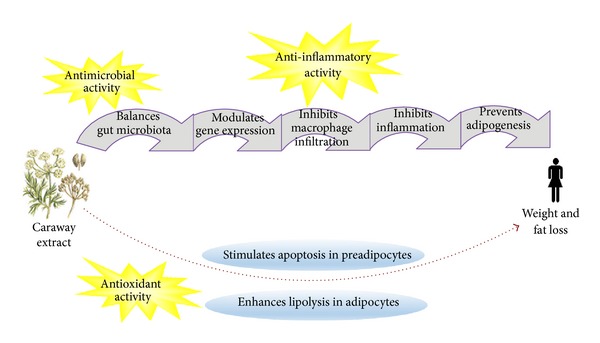
The possible metabolic actions of CE on the human body during weight loss.

Lowering weight and fat in the subjects in this study may be related to anti-microbial, anti-inflammatory, and antioxidant activities of caraway caused by some of the constituents in caraway, such as carvacrol (polyphenol) and unsaturated fatty acids (UFA) (Figure 2) [19]. These bioactive compounds may balance gut microflora (GM) which help in food digestion and absorption providing intestinal homoeostasis [40]. GM modulates gene expression in the human body involving the host physiology and metabolism, such as obesity mechanisms [41]. Carvacrol, together with UFA, inhibits the growth of pathogenic bacteria, and thus increases the proliferation of GM [42, 43]. In this process, ingredients probably modify GM through activating the expression of some specific genes involved in lipid metabolism inhibiting inflammation and adipogenesis [33, 44]. The balanced GM also inhibits infiltration of macrophages into obese adipose tissues leading to disruption in the conversion of preadipocytes to mature adipocytes, thereby preventing adipocyte differentiation and adipogenesis [45]. UFAs enhance the oxidation of fatty acids leading to lipolysis and fat loss [46, 47]. Caraway constituents also stimulate apoptosis in pre-adipocytes due to their antioxidant activity. They reduce adipose tissue mass through preventing adipogenesis and enhancing lipolysis in adipocytes [10, 48–50]. Further studies are suggested to investigate the effects of these potent components in reversing obesity in overweight and obese women at the molecular level. 

In this study, no changes were observed in the subjects' body water during the intervention, whereas body weight and fat mass were decreased, and muscle mass was increased. However, there were also significant changes in body composition in the placebo group, which shows that exercise did not have any interfering and/or synergistic effect on weight and fat loss. This implies that the favourable changes in body composition were probably associated with the bioactive compounds in CE and not necessarily with the physical activity, although it is recognized that there is likely a synergistic effect of exercise on weight and fat loss in the treatment group. It is plausible that the bioproducts formed during lipolysis were converted into muscle induced by physical activity, synergistically, and that the decrease in fat mass and the increase in lean mass are feasibly due to physiological adaptations to exercise [51–53].

To the best of our knowledge, this is the first clinical study to evaluate the effects of CE intake on body composition and anthropometric indices, combined with an exercise program, and examin to antiobesity effects of CE on overweight and obese women during a twelve-week intervention. In addition, this study had three other significant aspects. Firstly, even though the subjects' dietary habits were not modified, a significant weight loss was observed in the CE group, as compared with the placebo group. Secondly, this study was a triple-blind clinical trial which enhances the accuracy of the results and reduces potential bias in the findings. Some limitations of this study should be acknowledged. Firstly, these results are limited to an extract of caraway; therefore, further studies are required to find the anti-obesity effect of other methods of preparation for caraway seed oil extract. Secondly, as physical activity might have a synergistic effect on lowering weight [54], replicating this study on subjects without exercise is recommended. Thirdly, since this study was performed with adult females, in order to have a homogenous population and more reliable data, studies on the anti-obesity effects of CE in males, as well as in obese children, are suggested. Fourthly, in this study, obese or overweight subjects with medical complications, such as metabolic syndrome and cardiovascular disease (CVD), were specifically excluded. Future studies to examine the weight-lowering effects of caraway on overweight and obese patients having obesity complications are proposed. Finally, further studies are suggested to examine these results with different CE doses in order to establish more accurate dosing limitations.

## 5. Conclusions

From the above results and discussion, it can be concluded that caraway is helpful in the management of obesity because of its bioactive constituents. Although the mechanism of action of the active principle(s) remains to be determined at the molecular level, it is speculated to arise from a prebiotic effect of CE in the gut through balancing its GM growth. Efforts to provide information and understanding about the human use of this medicinal plant and make to the intake of caraway-containing natural and bioactive pharmaceuticals a sustainable dietary practice, along with physical activity, towards a healthy lifestyle, should be continued. 

## Figures and Tables

**Table 1:** Demographics of study participants randomized to the placebo or CE groups (*n* = 35).

Variables	Placebo	CE	*P* value
	Mean ± SD	Mean ± SD	
Age (years)	37.00 ± 7.90	37.23 ± 9.34	0.91
Height (cm)	158.20 ± 4.90	159.74 ± 6.22	0.25
Weight (kg)	74.88 ± 11.70	75.99 ± 11.84	0.70
Body mass index (BMI, kg/m^2^)	30.39 ± 4.69	29.24 ± 3.36	0.24
Bone mass (kg)	7.79 ± 1.11	8.05 ± 1.07	0.35
Sleep (hours/day)	7.87 ± 1.61	7.86 ± 1.29	0.97
Physical activity level (PAL, kcal/kg/day)	43.68 ± 2.48	44.39 ± 2.79	0.26
Basic metabolic rate (BMR, kcal/m^2^/hour)	1474.42 ± 123.46	1488.00 ± 154.43	0.69
Active metabolic rate (AMR, kcal/m^2^/hour)	2241.42 ± 216.27	2176.57 ± 260.47	0.30
Resting energy expenditure (REE, kcal)	1453.21 ± 133.26	1503.11 ± 127.37	0.15
Total daily energy expenditure (TDEE, kcal)	2236.06 ± 206.93	2308.33 ± 193.17	0.17

**Table 2:** Measured variables (mean ± SD) at baseline and after the three-month intervention period.

Variables	Week 0		Week 12	
	Placebo (*n* = 29)	CE group (*n* = 31)	Placebo (*n* = 29)	CE group (*n* = 31)
Body composition				
Weight (kg)	71.96 ± 10.66	76.86 ± 12.24	72.77 ± 10.84	75.0 ± 12.24*
Body mass index (BMI, Kg/m^2^)	28.34 ± 2.59	30.69 ± 4.69	28.50 ± 2.80	29.85 ± 4.70*
Body fat (BF, %)	33.82 ± 2.40	35.43 ± 3.60	34.04 ± 2.47	34.74 ± 3.74*
Body muscle (BM, %)	31.81 ± 1.27	31.42 ± 1.60	31.75 ± 1.29	31.61 ± 1.60*
Body water (BW, %)	48.34 ± 1.89	47.16 ± 2.63	48.12 ± 1.78	47.15 ± 2.67
Anthropometric indices				
Waist circumference (WC, cm)	91.34 ± 7.33	96.02 ± 10.21	91.21 ± 7.90	89.78 ± 8.64*
Waist-to-hip ratio (WHR)	0.87 ± 0.04	0.86 ± 0.06	0.87 ± 0.05	0.83 ± 0.05*
Paraclinical assessments				
Diastolic blood pressure (DBP, mmHg)	74.29 ± 6.0	75.48 ± 7.89	70.97 ± 7.60	75.9 ± 6.80
Systolic blood pressure (SBP, mmHg)	111.25 ± 10.33	112.74 ± 10.40	111.25 ± 9.49	113.39 ± 11.21
Heart rate (beats per minute)	75.21 ± 8.70	78.06 ± 9.11	74.46 ± 8.56	77.51 ± 8.11
Lipid profile				
Cholesterol (mg/dL)	183.33 ± 22.56	209.33 ± 29.87	190.38 ± 51.9	199.0 ± 25.1
Triglyceride (TG, mg/dL)	121.86 ± 41.49	112.81 ± 35.14	145 ± 50.4	124.43 ± 42.6
High density lipoprotein (HDL, mg/dL)	52.95 ± 9.87	55.90 ± 9.57	51.71 ± 7.7	56.71 ± 10.1
Low density lipoprotein (LDL, mg/dL)	106.73 ± 17.67	123.94 ± 28.65	110.77 ± 41.9	125.77 ± 25.9
Urine test				
Urine-specific gravity (USG, g/mL)	1.017 ± 0.006	1.021 ± 0.006	1.018 ± 0.005	1.022 ± 0.006

*p < 0.01 significantly different from baseline compared to placebo.
